# Effects of substrate stiffness on the viscoelasticity and migration of prostate cancer cells examined by atomic force microscopy

**DOI:** 10.3762/bjnano.13.47

**Published:** 2022-06-28

**Authors:** Xiaoqiong Tang, Yan Zhang, Jiangbing Mao, Yuhua Wang, Zhenghong Zhang, Zhengchao Wang, Hongqin Yang

**Affiliations:** 1 Key Laboratory of Optoelectronic Science and Technology for Medicine of Ministry of Education, Fujian Provincial Key Laboratory for Photonics Technology, Fujian Normal University, Fuzhou 350007, China; 2 Fujian Provincial Key Laboratory for Developmental Biology and Neurosciences, College of Life Sciences, Fujian Normal University, Fuzhou 350117, China

**Keywords:** actin cytoskeleton, atomic force microscopy, migration, prostate cancer cells, substrate stiffness, viscoelasticity

## Abstract

The stiffness of the extracellular matrix of tumour cells plays a key role in tumour cell metastasis. However, it is unclear how mechanical properties regulate the cellular response to the environmental matrix. In this study, atomic force microscopy (AFM) and laser confocal imaging were used to qualitatively evaluate the relationship between substrate stiffness and migration of prostate cancer (PCa) cells. Cells cultured on stiff substrates (35 kPa) undergone several interesting phenomena compared to those on soft substrates (3 kPa). Here, the stimulation generated by the stiff substrates triggered the F-actin skeleton to bundle its filaments, increasing the polarity index of the external contour of PCa cells. Analysis of AFM force–distance curves indicated that the elasticity of the cells cultured on 35 kPa substrates increased while the viscosity decreased. Wound-healing experiments showed that PCa cells cultured on 35 kPa substrates have higher migration potential. These phenomena suggested that the mechanical properties may be correlated with the migration of PCa cells. After actin depolymerisation, the elasticity of the PCa cells decreased while the viscosity increased, and the migration ability was correspondingly decreased. In conclusion, this study clearly demonstrated the relationship between substrate stiffness and the mechanical properties of cells in prostate tumour metastasis, providing a basis for understanding the changes in the biomechanical properties at a single-cell level.

## Introduction

Prostate cancer is a common malignancy of the male urinary tract and has become the second most threatening type of cancer in male patients after lung cancer [1–2]. Clinical data indicate that 90% of patients have a survival rate of more than 10 years if the prostate tumour is located in the prostate at the time of diagnosis and there are no distant metastases [3–4]. However, most patients with high-risk PCa have a poor prognosis or even clinical treatment failure due to the occurrence of distant metastases [5–6]. Therefore, the study of metastasis mechanisms is of great importance to the clinical management of PCa.

There are many factors that mediate tumour metastasis. Of these, the extracellular matrix (ECM) is most closely associated with tumour metastasis. The ECM is made of extracellularly secreted macromolecules. It not only contains a large number of biochemical factors, but also provides a suitable mechanical environment for cells, including physical signals such as substrate stiffness, hydrostatic pressure, shear stress, strain, pressure, and tension [7–9]. These mechanical factors play an important role in regulating normal cellular physiological functions and disease development. Studies have shown that solid tumorigenesis and metastasis are often accompanied by abnormal ECM cross-linking, remodelling, and increased tissue stiffness [10]. Peng et al. observed that substrate stiffness directly activates integrin β1 and adherent spot kinase, accelerates the maturation of focal adhesions, and induces a downstream cascade of intracellular signals in the RhoA/ROCK pathway, thereby promoting breast cancer cell motility [11]. Dai et al. found that high-stiffness matrices regulate the morphology of MG63 osteosarcoma cells, promote actin polymerization and nuclear accumulation of the cardiomyosin-related transcription factor A, induce epithelial-mesenchymal transition (EMT) in osteosarcoma cells, and thus promote osteosarcoma metastasis [12]. Differences in ECM stiffness do not only affect the development of breast cancer and osteosarcoma. Recent studies on pancreatic and hepatocellular carcinoma have also found that a stiffer ECM promotes EMT [13–14]. Therefore, the study of ECM stiffness has important implications for tumour metastasis.

For prostate cancer, the current clinical approach to improving the positive biopsy rate is to incorporate ultrasound elastography, whose imaging is based on the differences in stiffness between the lesion and the adjacent healthy tissue. It was found that an external environment with a high stiffness value promotes PC-3 cell migration and proliferation by inducing yes-associated protein and tafazzin (YAP/TAZ) nuclear localisation, suggesting that the behaviour of PCa cells is regulated by the external environment [15]. Traditional biological approaches to studying prostate cancer are based on molecular genetics and gene signalling. However, the cellular mechanistic properties that allow cells to express various biological functions have not been well appreciated [16]. In recent years, alterations in the physical properties of cells have been considered as a marker of malignant transformation of cancer cells [17–19]. Based on atomic force microscopy (AFM) measurements, our group found that the progression of prostate cancer tissue is related to its biomechanical properties (i.e., the higher the pathological grade of prostate cancer tissue, the less elastic and viscous it is) indicating that the mechanical properties of the tissue can predict the pathological grade of prostate cancer. This is consistent with the pathological diagnosis of transrectal ultrasound biopsy, suggesting that changes in the mechanical properties of prostate cancer tissue are closely related to tumour metastasis [20]. However, the relationship between the regulation of cellular behaviour by the extracellular environment and the mechanical properties of the cells themselves has not been discussed in detail.

In this study, polyacrylamide hydrogel substrates with different stiffness values (3–35 kPa) were prepared to simulate the stiffness of normal and prostate cancer tissues [21–23]. Combined with confocal microscopic imaging techniques and atomic force microscopic imaging, the changes in the mechanical properties of the cells themselves during cell migration were investigated by regulating the substrate stiffness. The results contribute to the understanding of the relationship between substrate stiffness and prostate cancer metastasis and possible regulatory mechanisms, which can further guide the study and treatment of cancer metastasis.

## Results and Discussion

### Effect of substrate stiffness on the migration of prostate cancer cells

**Figure 1:**
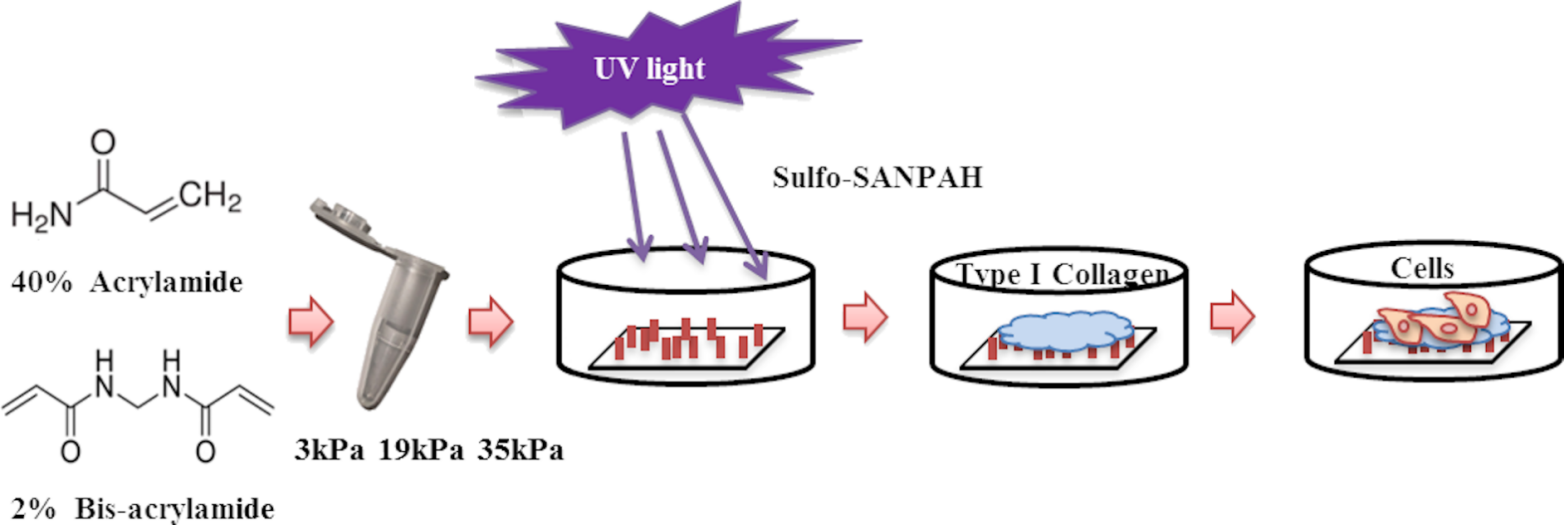
Schematic diagram of polyacrylamide hydrogel substrate preparation and cell culture process.

**Figure 2:**
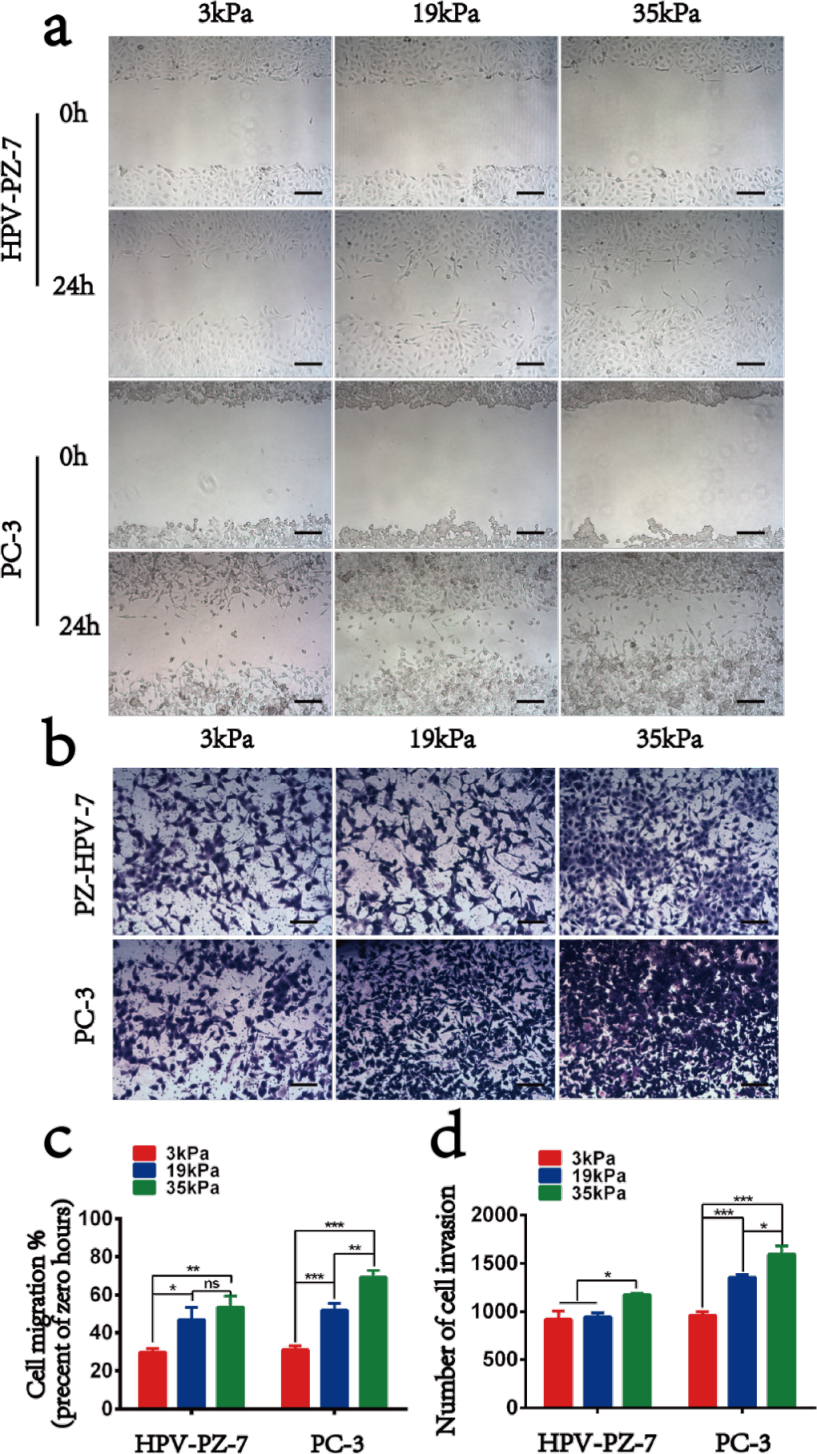
Effect of substrate stiffness on the migration of HPV-PZ-7 and PC-3 cells. (a) Analysis of cell wound healing. (b) Transwell analysis of the invasion ability. (c) Quantitative evaluation of the migration ability. (d) Quantitative evaluation of the invasion ability. Scale bar = 50 μm; “ns” means no difference, * represents the value of *P* < 0.05, ** represents the value of *P* < 0.01, and *** represents the value of *P* < 0.001.

In order to investigate the mechanical properties and metastatic ability of human prostate cancer cells in different external environments, polyacrylamide hydrogel substrates with adjustable stiffness were prepared by controlling the concentration of acrylamide and bisacrylamide on these gels (Figure 1). The stiffness values were 3 kPa (soft group) and 35 kPa (stiff group), representing normal prostate tissue and tumour tissue, respectively, and 19 kPa, an intermediate transition group. We first tested the toxicity of the hydrogel substrates to the cells and found that all three types of substrates were nontoxic to the cells and the cells were mostly active after 48 h of incubation (Supporting Information File 1, Figure S1). After culturing the cells on the substrates for 48 h, we measured the ability of the cells to migrate on the hydrogels for 24 h to ensure that the cells were in a stable state on the hydrogels (Figure 2a). We observed that the rate of cell migration on the hydrogel was stiffness dependent. As the stiffness of the substrate increased, the cell migration rate also increased. Specifically, compared to HPV-PZ-7 cells on stiff substrates (35 kPa), PC-3 cells moved in a sheet-like fashion and nearly closed the gap. Cells on intermediate substrates (19 kPa) had the next lowest migration capacity, and cells on soft substrates (3 kPa) had the lowest migration capacity (Figure 2c). Cell proliferation assays also revealed that both cell lines had a significantly greater ability to proliferate on stiff substrates, and that the proliferation of prostate cancer cells significantly increased with increasing substrate stiffness. (Supporting Information File 1, Figure S2a,b). The migration and proliferation assays were performed by culturing the cells on the substrates for 48 h, digesting them, and then inoculating them onto 6- and 96-well plates. This suggests that after 48 h the effect of the substrate on cells altered the internal mechanical properties of the cells and is no longer solely caused by the external physical environment. Invasion experiments revealed that cells on stiff substrates were more aggressive, with more cells crossing the upper chamber, compared to cells on other substrates (Figure 2b and Figure 2d). Soft substrates appear to give the cells a suitable attachment site, allowing for little movement. Their behaviour exhibited a lower correlation with the cellular substrate. Our study suggests that sclerosis of the extracellular matrix enhances the growth and viability of cancer cells and can promote their migration and invasion.

### Morphological analysis showed different cellular characteristics in different substrates

**Figure 3:**
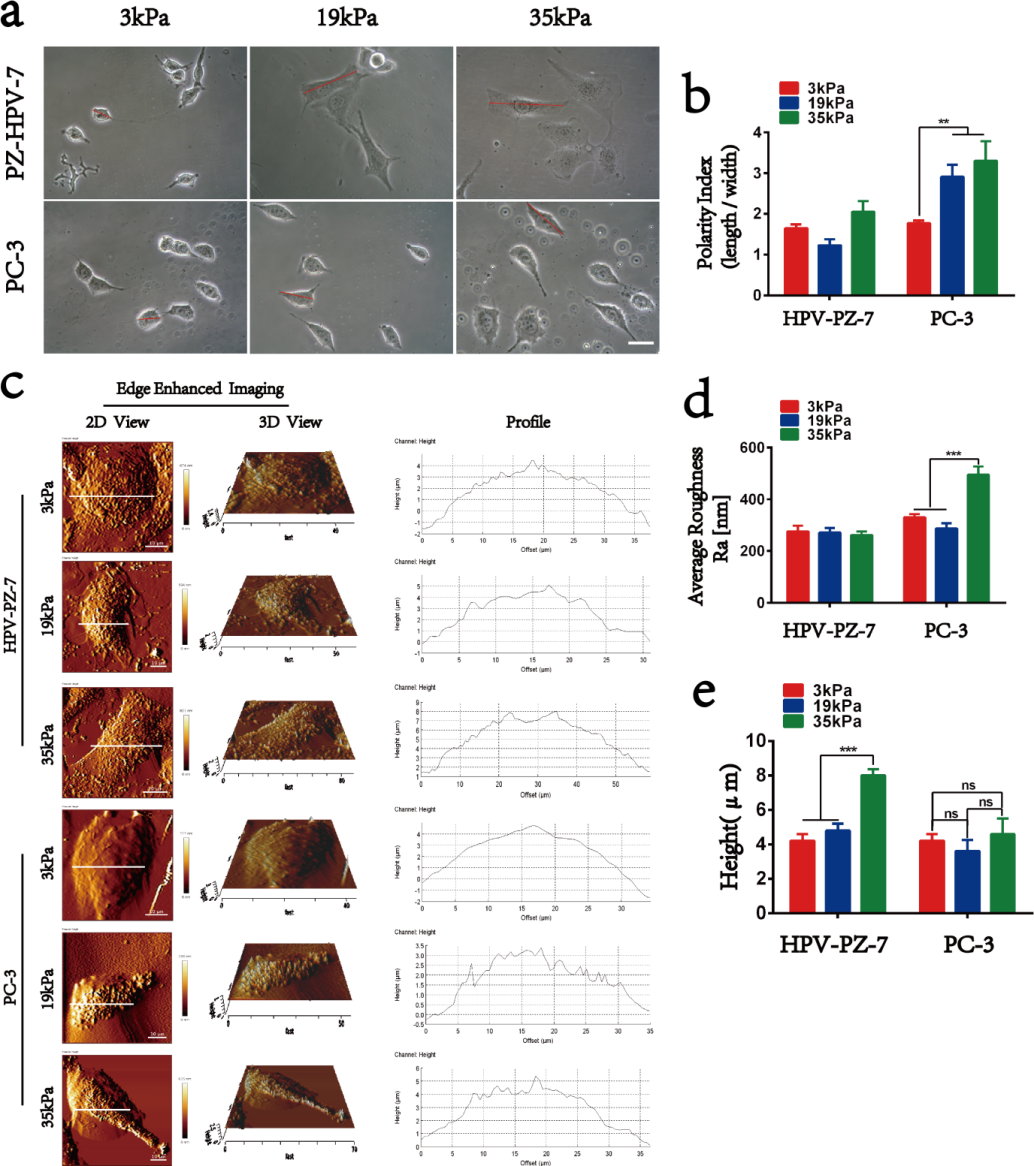
Morphological analysis showed different characteristics in different stiffness. (a) Phase-contrast microscopy imaging of PZ-HPV-7 and PC-3 cells on substrates with different stiffness, scale bar = 10 μm. (b) Quantitative statistical graph of cell polarity index (length/width). (c) Atomic force microscopy imaging of HPV-PZ-7 and PC-3 cells on substrates with different stiffness. From left to right, the edge-enhanced images of cells are displayed, including two-dimensional and three-dimensional imaging and cell contour maps. (d) Quantitative statistics of the average surface roughness (Ra). (e) Quantification plot of cell height; “ns” means no difference, ** *P* < 0.01, *** *P* < 0.001.

Differences in cell motility on hydrogels with different stiffness values are inevitably limited by the physical limitations of the hydrogel, which most visually affects the morphology of the cells. When HPV-PZ-7 and PC-3 cells were cultured on hydrogels of different stiffness values for 48 h, we observed through the analysis of phase-contrast microscopy images that most cells on stiff substrates were elongated and had a higher degree of cell expansion. In contrast, the cells on soft substrates were rounded and had a lower degree of cell expansion (Figure 3a). By measuring the cell surface area, perimeter, and polarity index, it was found that an increase in substrates stiffness was positively correlated with cell expansion. There was a significant difference between the surface area and perimeter of HPV-PZ-7 and PC-3 cells on stiff and soft substrates (Supporting Information File 1, Figure S3a,b). The polarity index showed that PC-3 cells had a higher polarity on stiff substrates, while HPV-PZ-7 cells were not sensitive to substrate stiffness (Figure 3b). The stiffer the hydrogel substrate, the greater the polarity index of the cancer cells. This suggests that the cell polarity index can be an indicator of how cells respond to the extracellular environment. This finding is consistent with previously reported results [24–25]. The significant polygonal shape of cells is widely believed to be associated with enhanced migration and invasion abilities. Of these, round cells spread more slowly, whereas morphologically elongated cells, due to their resemblance to fibroblasts, have prominent leading edges and retractable tails, and therefore exhibit greater flexibility [26]. The morphological imaging on the micro-/nanoscale of the cell membrane in extracellular environments with different stiffness values is shown in Figure 3c. The leading edge of the PC-3 cell membrane on stiff substrates is prominent, with a more pronounced ridge-like protrusion on the surface of the cell pseudopod. In contrast, the surface of PC-3 cells on soft substrates was smoother. In addition, both cell lines on the stiff substrate were observed to be taller than those on the other two substrates, with cell height values ranging from 1–4 μm. The height of the HPV-PZ-7 cells had the highest value on the stiff substrate, indicating that normal cells were largely noninvasive at this stiffness. In contrast, the height difference between PC-3 cells on stiff versus soft substrates was only approx. 1 μm and, therefore, not significant. This indicates that prostate cancer cells are not sensitive to the extracellular substrate stiffness (Figure 3e). All these phenomena suggest that a stiff extracellular environment contributes to the protrusion and extension of the leading edge of the cancer cells, as the formation of filamentous pseudopods is thought to be crucial for cell invasion [25–26]. Our results suggest that stiff substrates promote the protrusion of the leading edge of cancer cell membranes to guide cell motility. The average surface roughness of cells on extracellular environments with different stiffness is shown in Figure 3d. PC-3 cells showed a higher average surface roughness (Ra) on stiff substrates than on soft substrates, in contrast to HPV-PZ-7 cells, which did not exhibit this feature. Peak-to-valley ratio roughness (Rt) and root mean square surface roughness (Rq) also showed the same characteristics, see Supporting Information File 1, Figure S3c,d. Cell surface roughness is a quantitative measurement of the variability of cellular surface topography and serves as an indicator to assess the state of the cell (i.e., the greater the roughness, the greater the undulation of the cell surface topography [27]**)**. It can be involved in many cellular behaviours such as cell migration and adhesion and is an important indicator of the physiological state of the cells [28–29]. Thus, stiff substrates do promote migration of prostate cancer cells by altering their morphology, including cellular polarity index, filamentous pseudopods, and surface roughness.

### Cytoskeletal microfilaments of prostate cancer cells respond to changes in substrate stiffness

**Figure 4:**
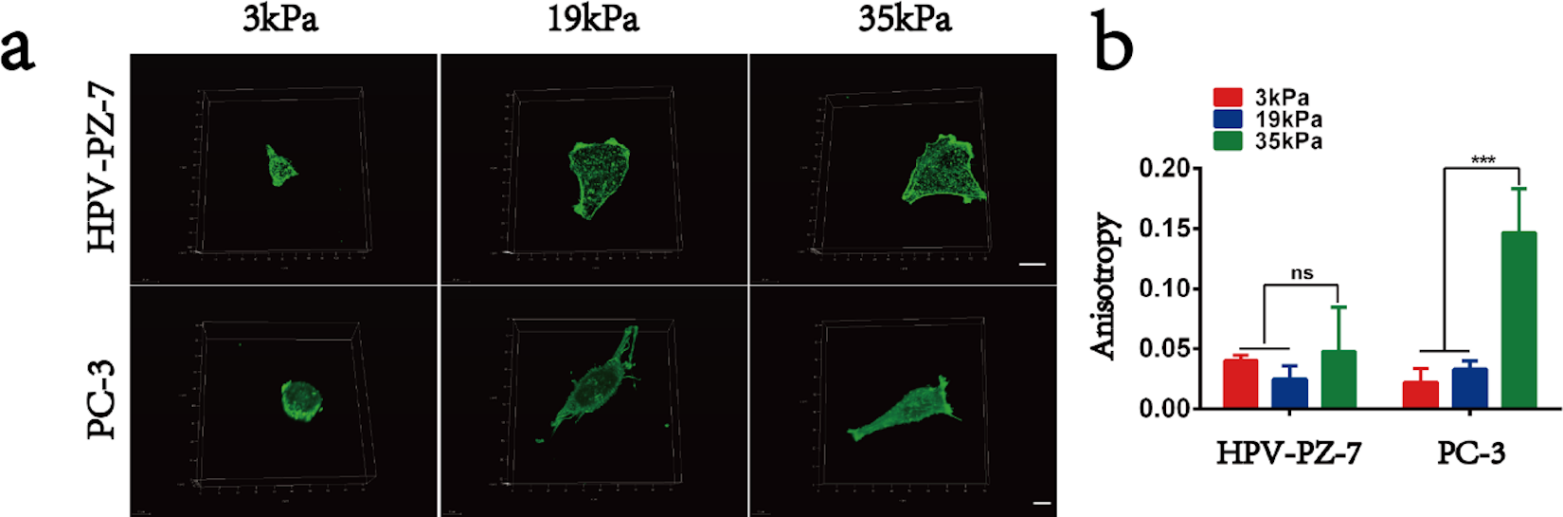
Cytoskeletal microfilaments respond to changes in substrate stiffness on prostate cancer cells. (a) Fluorescence images of HPV-PZ-7 and PC-3 cytoskeletal microfilaments of cells seeded on substrates with different stiffness. The scale bar of HPV-PZ-7 cells is 20 μm and the scale bar of PC-3 cells is 10 μm. (b) Anisotropy quantification maps of the fibrillar structure of HPV-PZ-7 and PC-3 cells on substrates with different stiffness. FibrilTool, an ImageJ plug-in, was used to quantify the fibrillar structure in the original cytoskeleton images. Anisotropy (score between 0 and 1): 0 for no order (purely isotropic arrays) and 1 for perfectly ordered arrays (i.e., parallel fibrils, purely anisotropic arrays); “ns” means no difference, *** *P* < 0.001.

We further considered how the role of cell sensing substrates makes cancer cells more capable of migrating on stiff substrates. The distribution and content of F-actin within the cells was examined using fluorescent isothiocyanate (FITC)-labelled phalloidin to analyse the distribution and local directional changes of filamentous actin. It could be seen that the microfilaments of PC-3 cells were densely arranged and ordered on the stiff substrate. The quantified anisotropy of cytoskeletal microfilaments of PC-3 cells was significantly higher than that of HPV-PZ-7 cells. In addition, PC-3 cells were found to have distinct filamentous pseudopods on stiff substrates (Figure 4a,b and Supporting Information File 1, Figure S4a), which was consistent with the results obtained from micro-/nanoscale imaging of the cell membrane. We observed that prostate cancer cells exhibit a strong migration ability by sensing changes in the extracellular environment through actin polymerization and filamentous pseudopods. This is because the role of actin polymerisation in cell adhesion structure formation, maturation, and myosin contraction has been found to be an important factor in cell migration [30–32]. Cytoskeletal myosins drive cell contraction and enhance the longitudinal tension of the cell causing the cell to contract in the direction of cell migration, resulting in the displacement of the entire cell [33–35].

### Mechanical properties of prostate cancer cells respond to substrate stiffness

**Figure 5:**
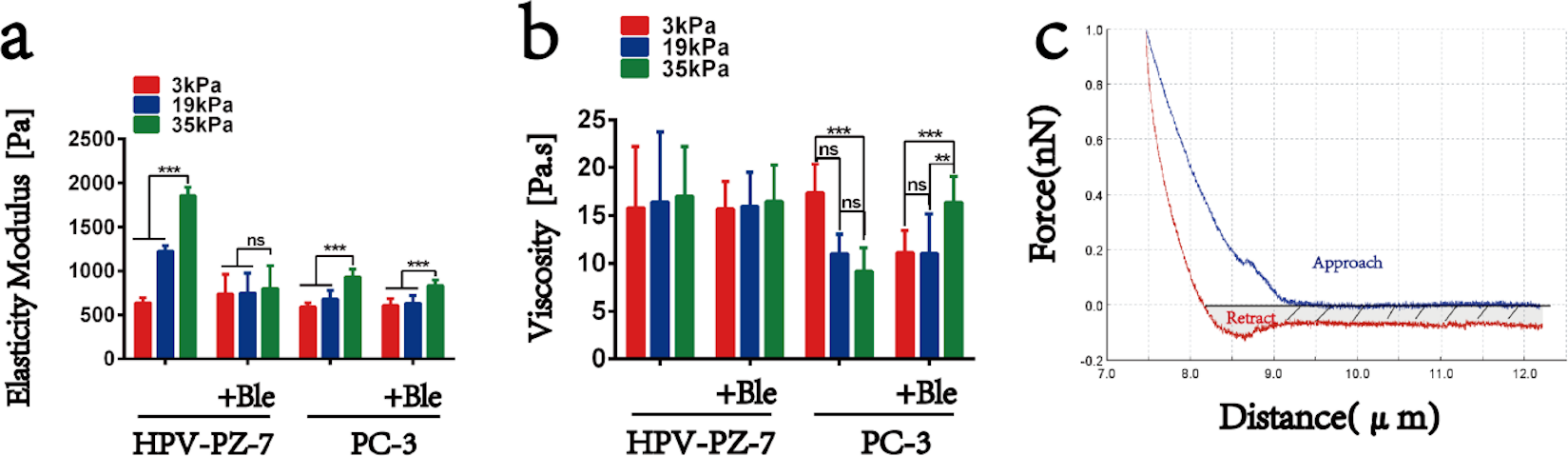
Mechanical properties respond to the effect of substrate stiffness on prostate cancer cells. (a) The average elasticity value of HPV-PZ-7 and PC-3 cells on substrates of different stiffness with or without blebbistatin. (b) The average viscosity value of HPV-PZ-7 and PC-3 cells on substrates of different stiffness with or without blebbistatin. (c) Force–distance graph of cells; “ns” means no difference, ** represents the value of *P* < 0.01, *** represents the value of *P* < 0.001.

The previous experiments have shown that stiff substrates promote prostate cancer cell migration by altering cell morphology and actin distribution, so what are changes in the mechanical properties of the cells themselves in response to the action of the substrate? Atomic force microscopy experiments at the nanoscale were used to measure changes in the elasticity of live cells in situ and to quantify the mechanical response of HPV-PZ-7 and PC-3 cells to the extracellular environment with different stiffness values. To overcome the effect of different indentation depths on elasticity, we measured the cells in the same area and at the same force. PC-3 cells were found to have significantly lower elasticity values than HPV-PZ-7 cells, indicating that prostate cancer cells are less stiff than normal cells. This is consistent with previous reports on other cells [36–37]. Moreover, we found that the higher the substrate stiffness, the higher the cellular elasticity values, suggesting that the spreading of the cells has an effect on their mechanical properties, with cells that are adequately spread being stiffer than those that are less spread (Figure 5a and Supporting Information File 1, Figure S6a). Furthermore, considering the rheological behaviour of the living cells themselves (i.e., the energy dissipated during the downward pressure of the probe to deform and recover the cells from deformation [38–39]) the energy dissipation was mainly caused by cell adhesion, which was a certain separation between the approach and retraction curves (Figure 5c). The results showed a negative correlation between viscosity values and substrate stiffness in PC-3 cells: the higher the substrate stiffness, the lower the cell viscosity. Conversely, HPV-PZ-7 cells did not show this characteristic (Figure 5b and Supporting Information File 1, Figure S6b). The physical quantity expressed by viscosity is inversely proportional to mobility. The lower the viscosity, the more mobile the cell is, and the mobility of the cells is positively correlated with their ability to invade. Wullkopf et al. [40] found that, in a 3D breast cancer model, an increased viscosity might actually aid cells at an invasive front. Our study firstly shows that substrate stiffness could affect the migration and invasion of prostate cancer cell through cellular mechanical properties. Thus, prostate cancer cells respond to the effects of their extracellular environment by altering their mechanical properties (both elasticity and viscosity) and thus their ability to migrate. Changes in the mechanical properties of the cells themselves are closely related to differences in their extracellular environment, which are determined by the tension and structural organisation of the cytoskeleton [33–34]. From the results of our study it is clear that the control cells are more sensitive to stiffness in terms of their increased elasticity compared to PCa cells. The reverse is true for viscosity. It is shown that normal cells respond to stiff substrates by increasing elasticity without affecting viscosity in order to prevent shape changes (or flow) which leads to cell movement. And cancer cells, despite stiffening to a certain extent, lower their viscosity rather than maintaining it, leading to cell movement and invasion. Kahn et al. [41] found that softening of prostate cancer cell nuclei rather than fluidization perhaps is a better predictor of metastatic potential using flowing suspended cells through a confined space. Whether this could be a better index of metastatic potential for prostate cancer, it would need further studies. Our results seem to indicate that the cytoskeleton plays an important role in mediating migration capacity in different extracellular environments.

### Substrate stiffness affects PCa cell migration through changes in cytoskeleton microfilaments and mechanical properties

To further determine whether substrate stiffness affects prostate cancer cell migration by acting upon cytoskeletal microfilaments, we treated the cells with blebbistatin, a non-muscle myosin type II ATPase inhibitor [42]. A treatment with 20 µM of blebbistatin for 30 min revealed disorganised microfilament bundles on stiff substrates and weaker fluorescence intensity in PC-3 cells rather than in non-blebbistatin-treated cells (Supporting Information File 1, Figure S4b–d). The Young's modulus of HPV-PZ-7 and PC-3 cells on stiff substrates was reduced after blebbistatin treatment, with no effect on soft substrates. However, prostate cancer cells on stiff substrates continued to exhibit high elasticity (Supporting Information File 1, Figure S6c and Figure 5a). The viscosity data showed an increase in viscosity values for PC-3 cells on stiff substrates after blebbistatin treatment (Supporting Information File 1, Figure S6d and Figure 5b). The results indicate an overall decrease in cell stiffness after blebbistatin treatment. However, the 35 kPa stiffness substrates continued to show a mechanical response given the possible duration effect of blebbistatin. The results of migration and invasion assays showed that the migration and invasion abilities of both cells on substrates with different stiffness were reduced compared to those without blebbistatin treatment (Supporting Information File 1, Figure S5a–d). The results show that when we inhibit actin polymerisation with an actin-binding protein inhibitor, the actin fibrils fail to polymerise into bundles and the prostate cancer cells themselves have reduced elasticity values and increased viscosity values, thus reducing the migration ability of the prostate cancer cells. Non-muscle myosin II B (NMIIB) has been reported to regulate the generation and distribution of intracellular traction during basal stiffness-induced cell migration, thereby influencing cell motility by altering cell adhesion to the extracellular matrix [43]. Our study demonstrates that the altered viscosity of cancer cells in response to stiff substrates is myosin-dependent.

## Conclusion

To the best of our knowledge, the present study qualitatively evaluated for the first time the association between viscoelastic properties measured by atomic force microscopy with migration of PCa cells on substrates with different stiffness. On stiff substrates, the F-actin cytoskeleton organized into bundles and increased the elasticity while the viscosity of PC-3 cells decreased to enhance their migration ability. Furthermore, the results of the treatment with the myosin-binding protein inhibitor suggested that the response of PCa cells to the extracellular matrix may have been caused by differences in F-actin cytoskeleton organization, leading to changes in the mechanical properties of the cells. Since mechanical properties measured at the nanoscale can be used as an indicator of cancer cell migration, this study may provide a new approach to investigate the migration mechanism of PCa cells in addition to cytoskeleton-mediated migration of PCa cells, which can be useful for cancer therapeutic drug screening and development.

## Experimental

### Cell culture

PZ-HPV-7 normal human prostate epithelial cells and PC-3 prostate cancer cells were purchased from Shanghai ATCC cell bank. PZ-HPV-7 and PC-3 cells were cultured in Dulbecco's Modified Eagle’s Medium containing 10% of fetal bovine serum (FBS) and 1% of penicillin/streptomycin solution and Ham's F-12K medium containing 10% of FBS and 1% of penicillin/streptomycin solution, respectively. In order to allow for the acclimatisation of cells to different substrates, cells were inoculated and cultured on hydrogel substrates with stiffness values of 3, 19, and 35 kPa. Next, cells were digested with 0.25% trypsin after 48 h and continued in the hydrogel on the same substrate. This was done for three repeated passages. Cells were digested with trypsin and counted before other experiments were carried out [44].

### Preparation of polyacrylamide (PAA) gel substrates

The preparation of polyacrylamide hydrogels with different stiffness referred to the experimental method of Justin R. TSE [45]. In a nutshell, the cover glass with 25 mm diameter was uniformly covered with a thin layer of 0.1 M NaOH and dried. After 5 min of activation with 3-aminopropyl triethoxysilane (ServiceBio, China), sterile water was use to wash the activated coverslips. The treated glass slides were immersed into a 0.5% glutaraldehyde solution, placed at room temperature for 30 min, and then taken out to air dry. The slides were treated with dimethyldichlorosilane for 5 min, washed, and dried. Acrylamide (40%) and bis-acrylamide (2%, Sigma-Aldrich) were mixed in at a certain proportion, followed by the addition of 10% ammonium persulfate and 0.1% TEMED. After the mixture was fully mixed, 25 μL of this mixture was absorbed onto the treated glass slide, and the treated cover glass was quickly covered. After the gel was cross-linked for about 20 min, the cover glass was lightly removed, placed into sterile water, and stored at 4 °C. The stiffness of the hydrogels with different proportions were shown in Table 1. Next, the gels were coated with a sulfo-SANPAH solution and activated in UV light at 365 nm for 10 min. These gels were then coated with type I collagen (0.1 mg·mL^−1^) and incubated overnight at 4 °C. On the second day, the excess collagen solution was removed with sterile water, and then HPV-PZ-7 and PC-3 cells were inoculated onto PPA substrates with different stiffness for 12, 24, 48, and 72 h. The schematic diagram regarding this process is shown in Figure 1a.

**Table 1:** Composition ratio of polyacrylamide hydrogel substrates with different stiffness.

Sample	40% acrylamide (mL)	2% bis-acrylamide (mL)	H_2_O	elastic modulus (kPa)

soft	1.25	0.50	8.25	3.15 ± 0.85
medium	2.00	1.32	6.68	19.66 ± 1.19
stiff	2.50	1.50	6.00	34.88 ± 2.65

### Cell death assay

Cells were stained with calcein-AM and propidium iodide (PI) to label live and dead cells, respectively, for analysis. HPV-PZ-7 and PC-3 cells (1 × 10^6^ cells/mL) were cultured on PAA hydrogels with different stiffness for 12, 24, 48, and 72 h. The cells were then collected by digestion and centrifugation and the assay buffer was used to rinse the cells three times to remove residual esterase activity. Next, the cells were incubated at 37 °C for 15 min in assay buffer containing calcein-AM (1 μM) and PI (3 μM) and then imaged with a laser confocal microscope.

### Cell migration and invasion assays

To assess cell migration, cells were cultured on PAA gels with different stiffness for 48 h, removed by trypsinization, and seeded at a density of 2 × 10^6^ cells/mL in six-well plates to a confluent monolayer. A 10 µL pipet tip was used to scrape the cell monolayer in a straight line to create an empty gap. The debris was then removed by washing the cells three times with PBS and then the wells were replenished with 2 mL of fresh medium without serum. Then, the cells were imaged immediately and 24 h after the scratch.

For the quantification of the wound-healing assay, three locations in the image scratch were randomly selected, the width of cell scratch was measured, and the average value was calculated. The cell migration index (Im = (G0−G1)/G0·100%) was used to represent the speed of cell migration. G0 referred to the scratch width at 0 h (i.e., immediately after scratch) and G1 referred to the scratch width 24 h after the scratch.

The invasion assays were performed according to [11]. Cells were cultured on PAA gels with different stiffness for 48 h, removed by trypsinization, and then seeded into the upper compartment (2 × 10^4^ cells/well) of a transwell chamber (Corning, USA) with serum-free medium. The lower compartment of the transwell chamber had culture medium supplemented with serum. After incubation for 24 h, cells in the upper wells that did not invade were gently removed with cotton swabs. Cells that had passed through the membrane to the lower surface were fixed with 4% paraformaldehyde for 15 min and then stained using crystal violet for 10 min. Images were obtained using an inverted optical microscope (Nikon ECLIPSE TS100, Japan). These data were quantified using the ImageJ software.

### Cell proliferation assay

The proliferation of HPV-PZ-7 and PC-3 cells on substrates with different stiffness was detected by the CCK-8 assay. The cells were incubated on PAA hydrogels with different stiffness for 48 h, digested, and inserted into 96-well plates at a density of 1 × 10^4^ cells per well. The cells were incubated for 12, 24, 48, and 72 h. The cells were then treated with 10 µL of the CCK-8 reagent (Sigma-Aldrich)/100 µL medium for 1 h. The optical density (OD) values were then evaluated at 450 nm using a microplate reader (Bio-Rad).

### Cell morphology assay

HPV-PZ-7 and PC-3 cells were inoculated onto PAA hydrogel substrates with different stiffness at a density of 1 × 10^4^ cells/mL for 48 h. Then, the cell morphology of the substrates with different stiffness was obtained by using an inverted optical microscope (Nikon ECLIPSE TS100, Japan). The Illustrator software was used to analyse cell morphology and size.

### Atomic force microscopy

The elastic moduli of cells (2 × 10^4^ cells/mL) that were cultured on PAA gels with different stiffness for 48 h were determined by an AFM setup (JPK NanoWizard III, Germany) equipped with an inverted optical microscope (Leica, Germany). The measurements were performed at 37 °C and the temperature was maintained by a Petri dish heater (JPK instrument, Berlin Germany) for 2 h to prevent damage to the cells. Before the experiment, the thermal noise method was used to adjust the cantilever spring constant, and then the experiment was carried out in contact mode. The AFM probe (MLCT probe, Bruker, USA) slightly contacted the cell surface and a constant force was maintained. An indentation area of 3 μm × 3 μm was selected at the nuclear region where 36 force curves were recorded for each cell in force spectroscopy mode. The indentation force of 1 nN, spring constant values of 0.01 N·m^−1^, Z length of 5 μm, and an approach speed of approximately 2 μm·s^−1^ were employed in all AFM experiments. The same parameters were maintained for all cells measured. The elastic modulus was acquired based on the Hertz model and the viscosity was calculated using the method proposed by the Rebelo’s group which combines the traditional method with the AFM retraction force curve [46–47]. This method can be used to measure the Young's modulus and calculate cellular viscosity directly from the measured force spectrum.

For morphometric analysis, cells (2 × 10^4^ cells/mL) cultured on PAA gel with different stiffness for 48 h were fixed in 4% paraformaldehyde for 15 min and washed with 2 mL of PBS. Then, the cells were observed in QI working mode with Setpoint 1 nN, Z length of 2000 nm, and pixel time of 50 ms. The topography scanning at each pixel position (128 × 128) of the selected area (50 μm × 50 μm) was done to obtain high-resolution surface topography features of cells. The profiles of cells were extracted from topographic images using the Image J software. Tridimensional reconstitution of topographic images was performed using the JPK software. Likewise, the same parameters were maintained for all cells measured.

### Confocal microscopy

Cells (5 × 10^4^ cells/mL) were plated onto polyacrylamide gel substrates and fixed in 4% paraformaldehyde for 15 min at room temperature. Then, the cells were washed with PBS, permeabilised with 0.1% Triton X-100 for 10 min, washed again with PBS, and incubated with ActinGreen (KeyGEN, BioTECH) for 40 min in the dark. The samples were washed with PBS and fluorescence images were obtained using an inverted optical microscope (Leica, Germany) with 488 laser lines. The obtained images were linearly analysed and pseudo-coloured using the ImageJ analysis software.

### Blebbistatin treatment assay

To inhibit myosin II, cells were incubated with the corresponding medium containing 20 μM of blebbistatin (GlpBio, USA) for 30 min prior to the measurements, and a 10 μM working solution was prepared with DMSO. DMSO was added to the corresponding medium as a control. The cells were washed three times with PBS and incubated in fresh medium for 30 min before the measurements.

### Statistical analysis

All representative qualitative data were replicated in at least three separate biological replicates. Student’s *t*-test and one-way ANOVA analyses were performed using the GraphPad Prism 6.0 software, as indicated in the captions of the figures in the main manuscript as well as in the Supporting Information. The values are presented as mean ± standard deviation of multiple measurements and statistical significance was considered at *P* < 0.05.

## Supporting Information

Figure S1: The toxic effect of polyacrylamide hydrogel substrates on prostate cancer cells. Figure S2: The effect of substrate stiffness on cell proliferation. Figure S3: The effect of substrate stiffness on cell morphology. Figure S4: The effect of blebbistatin on PCa cytoskeleton microfilaments. Figure S5: The effect of blebbistatin on PCa cell migration. Figure S6: Mechanical properties respond to the effect of substrate stiffness.

File 1 Additional figures.
