# Supplementary material for: Effects of substrate stiffness on the viscoelasticity and migration of prostate cancer cells examined by atomic force microscopy
Source: Beilstein J Nanotechnol. 2022 Jun 28;13:560–9. doi: 10.3762/bjnano.13.47 (PMC9263554; doi:10.3762/bjnano.13.47)
Supplement: File 1 — Additional figures. [file Beilstein_J_Nanotechnol-13-560-s001.pdf]

BEILSTEIN JOURNAL OF NANOTECHNOLOGY

Supporting Information

for

Effects of substrate stiffness on the viscoelasticity and migration of prostate cancer cells examined by atomic force microscopy

Xiaoqiong Tang, Yan Zhang, Jiangbing Mao, Yuhua Wang, Zhenghong Zhang, Zhengchao Wang and Hongqin Yang

Beilstein J. Nanotechnol. **2022,** *13,* 560–569. doi:10.3762/bjnano.13.47

Additional figures

License and Terms: This is a supporting information file under the terms of the Creative Commons Attribution License (https://creativecommons.org/licenses/by/4.0). Please note that the reuse, redistribution and reproduction in particular requires that the author(s) and source are credited and that individual graphics may be subject to special legal provisions.
The license is subject to the *Beilstein Journal of Nanotechnology* terms and conditions: (https://www.beilstein-journals.org/bjnano/terms)

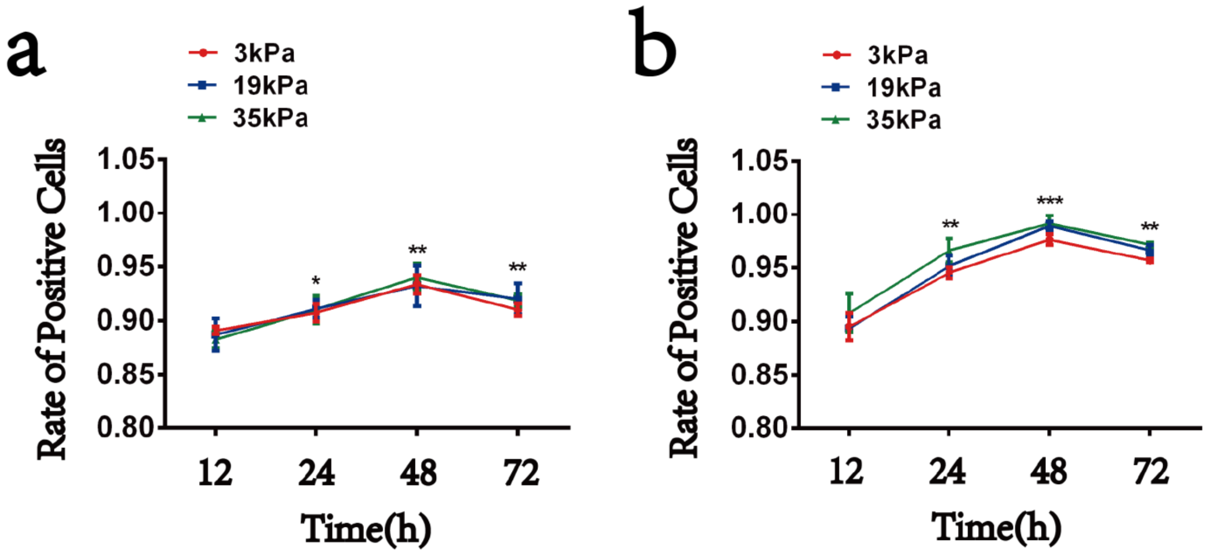

Figure S1: The toxic effect of polyacrylamide hydrogel substrates on prostate cancer cells. (a,b) Ratio of Calcein-AM/PI labelled HPV-PZ-7 and PC-3 cells, respectively.

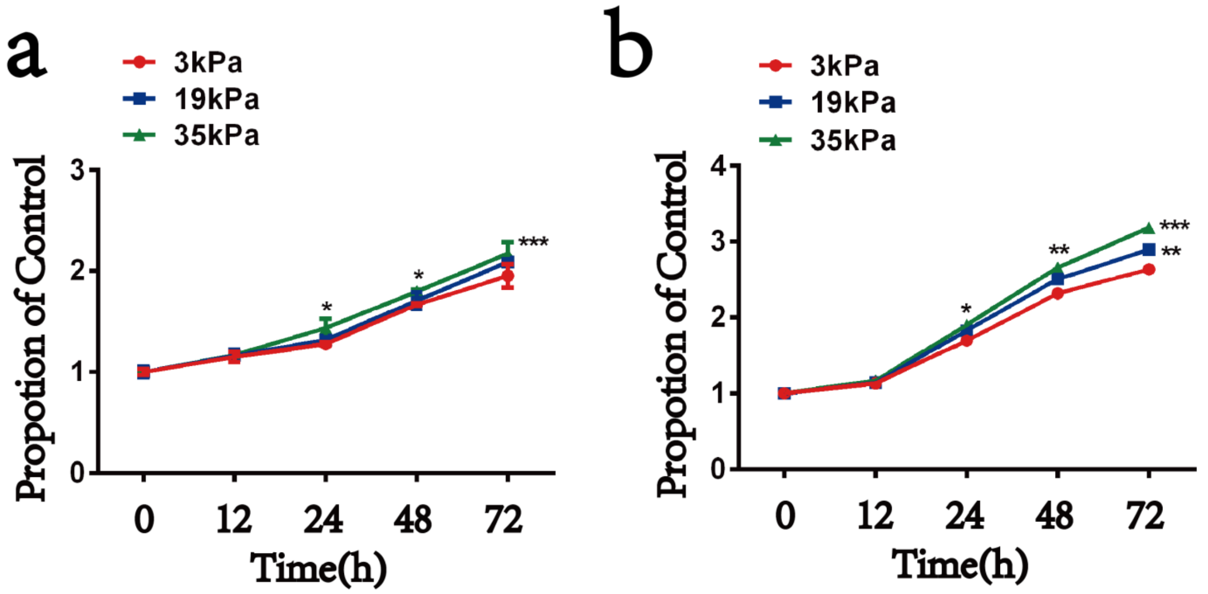

Figure S2: The effect of substrate stiffness on cell proliferation. (a,b) CCK-8 detects proliferation of HPV-PZ-7 and PC-3 cells. The proliferation rate of cells plated on 19 and 35 kPa was compared to that of cells plated on at 3 kPa substrates.

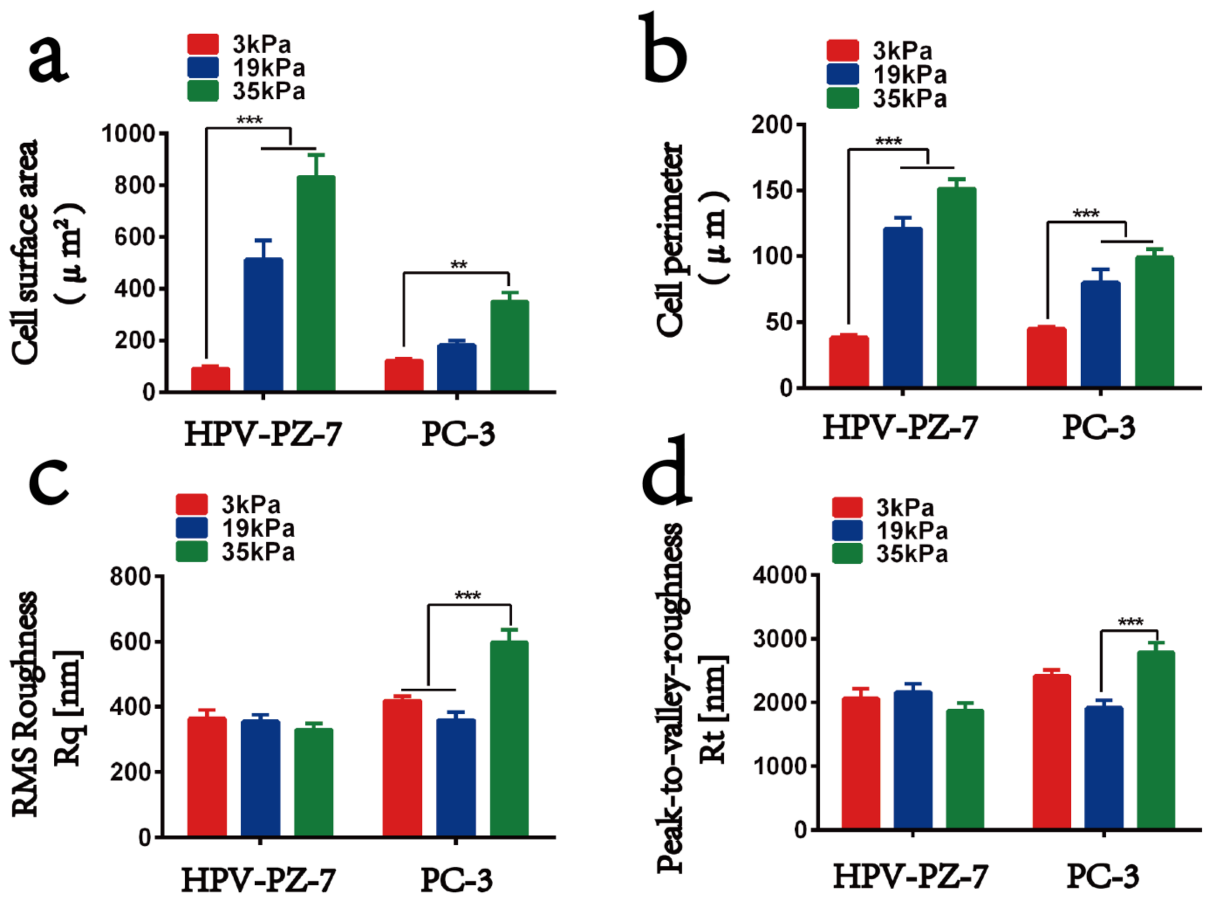

Figure S3: The effect of substrate stiffness on cell morphology. (a) Phase-contrast microscopy imaging of PZ-HPV-7 and PC-3 cells on different stiffness substrates were used to obtain measurements of cell area. (b) Measurements of cell perimeter. (c) Atomic force microscopy imaging of HPV-PZ-7 and PC-3 cells on substrates with different stiffness was used to obtain measurements of the root mean square surface roughness (Rq). (d) Measurements of peak-to-valley ratio roughness (Rt). ** $p < 0.01$, *** $p < 0.001$.

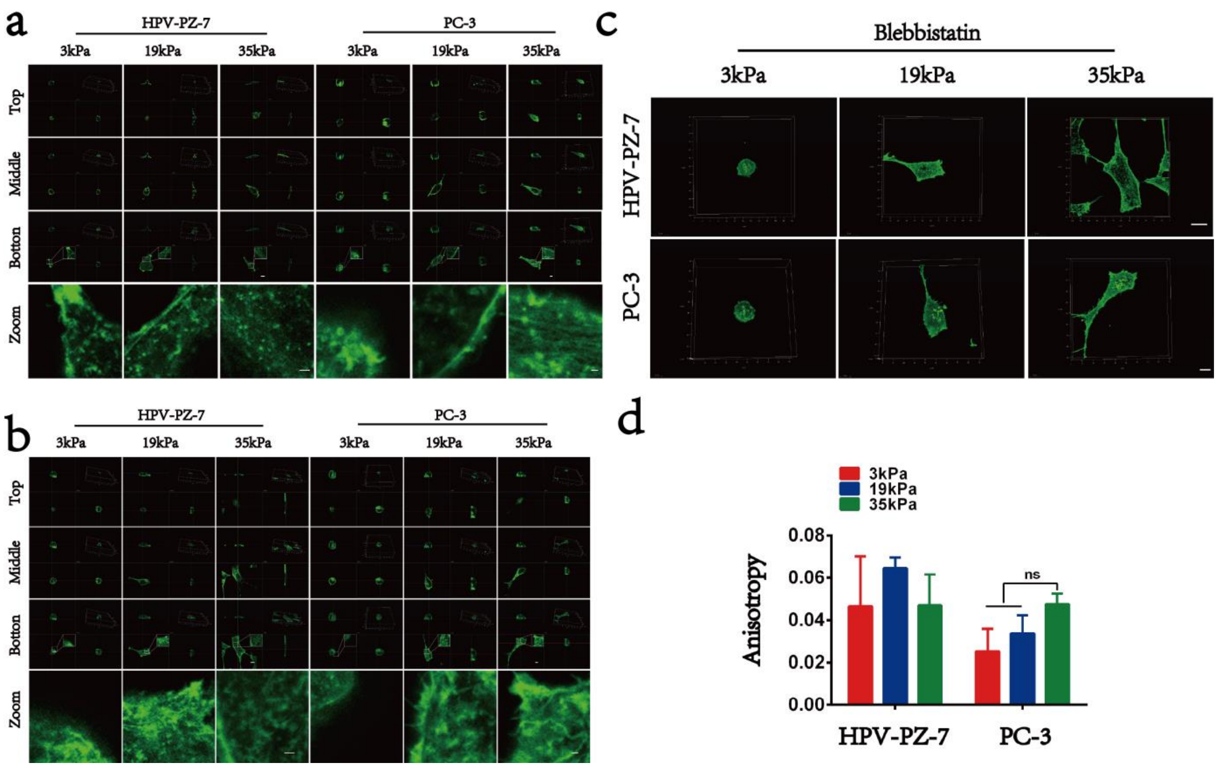

Figure S4: The effect of blebbistatin on PCa cytoskeleton microfilaments. (a) Three-dimensional fluorescence images of cytoskeletal microfilaments of HPV-PZ-7 and PC-3 cells on substrates with different stiffness. The zoomed image in the top right corner is a 2.5× enlargement of an area taken from the original image with a size of 2.2 × 2.2. The scale bar of HPV-PZ-7 cells is 20 μm and the scale bar of PC-3 cells is 10 μm. (b) Three-dimensional fluorescence images of cytoskeletal microfilaments of HPV-PZ-7 and PC-3 cells on substrates with different stiffness after blebbistatin treatment. (c) The fluorescence images of HPV-PZ-7 and PC-3 cytoskeletal microfilaments on substrates with different stiffness after blebbistatin treatment; the scale bar of HPV-PZ-7 cells is 20 μm, and the scale bar of PC-3 cells is 10 μm. (d) Anisotropy quantification maps of the fibrillar structure of HPV-PZ-7 and PC-3 cells on substrates with different stiffness after blebbistatin treatment; “ns” means no difference.

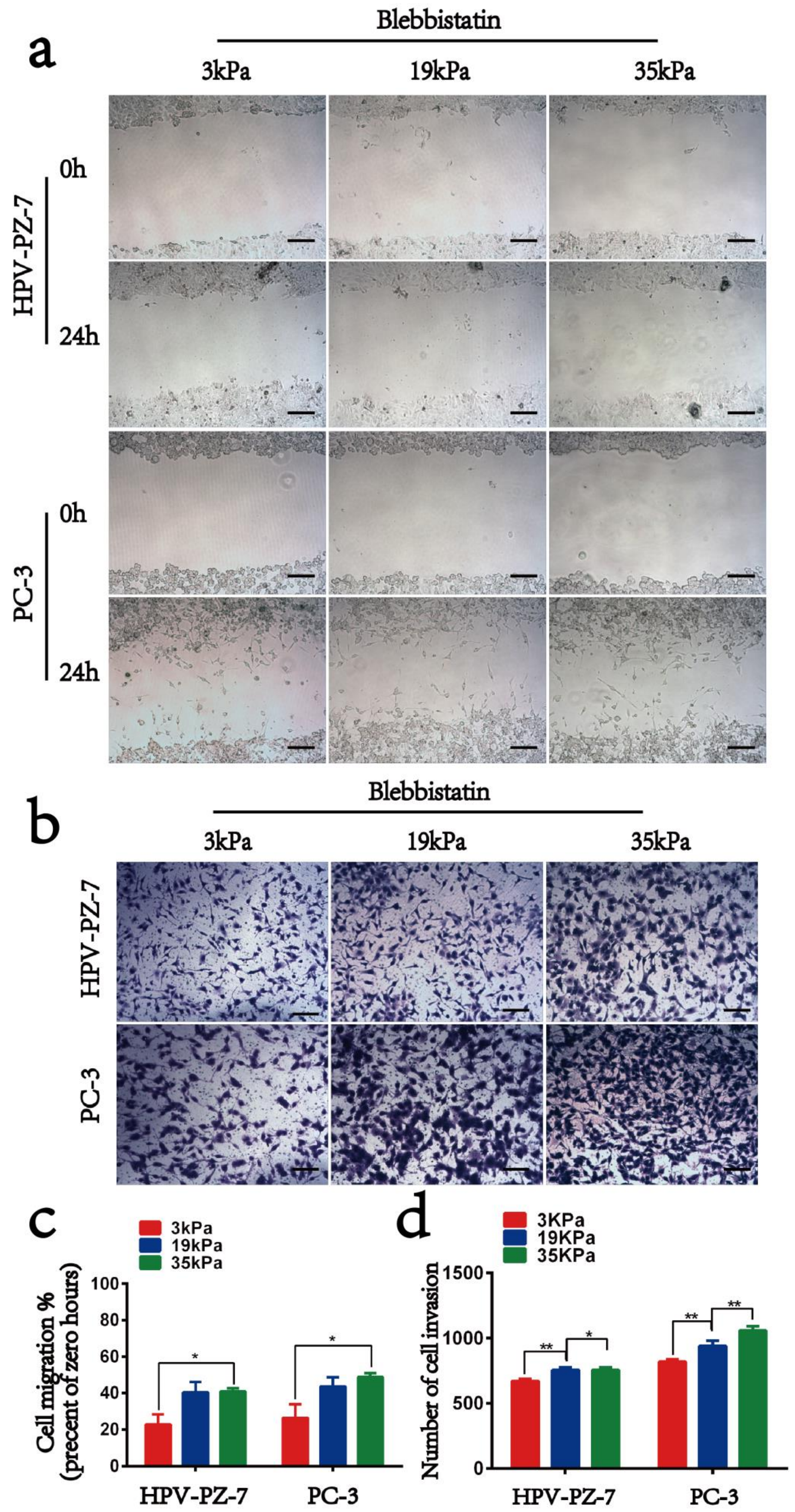

Figure S5: The effect of blebbistatin on PCa cell migration. (a) Cell wound healing analysis of the migration ability of HPV-PZ-7 and PC-3 cells on substrates with different

stiffness after blebbistatin treatment. (b) Analysis of transwell invasion ability of HPV-PZ-7 and PC-3 cells on substrates with different stiffness after blebbistatin treatment. (c) Quantitative analysis of the migration ability of HPV-PZ-7 and PC-3 cells on substrates with different stiffness after blebbistatin treatment. (d) Quantitative analysis of the invasion ability of HPV-PZ-7 and PC-3 cells on substrates with different stiffness after blebbistatin treatment. * $P < 0.05$, ** $P < 0.01$, *** $P < 0.001$.

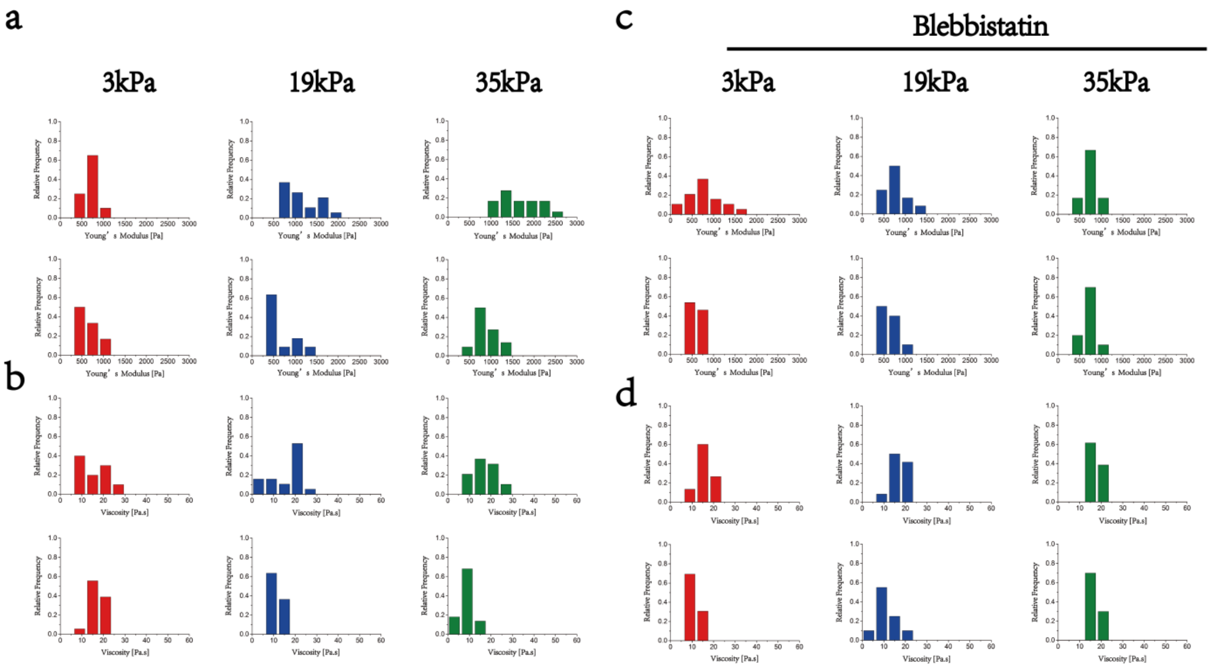

Figure S6: Mechanical properties respond to the effect of substrate stiffness on prostate cancer cells. (a) Histograms of the elastic frequency distribution of HPV-PZ-7 and PC-3 cells on substrates with different stiffness. (b) Histograms of the viscosity frequency distribution of HPV-PZ-7 and PC-3 cells on substrates with different stiffness. (c) Histogram of elastic frequency distribution of HPV-PZ-7 and PC-3 cells on substrates with different stiffness after blebbistatin treatment. (d) Histogram of the viscosity frequency distribution of HPV-PZ-7 and PC-3 cells on substrates with different stiffness after blebbistatin treatment.
